# Plant biomass and soil organic carbon are main factors influencing dry-season ecosystem carbon rates in the coastal zone of the Yellow River Delta

**DOI:** 10.1371/journal.pone.0210768

**Published:** 2019-01-14

**Authors:** Yong Li, Haidong Wu, Jinzhi Wang, Lijuan Cui, Dashuan Tian, Jinsong Wang, Xiaodong Zhang, Liang Yan, Zhongqing Yan, Kerou Zhang, Xiaoming Kang, Bing Song

**Affiliations:** 1 Beijing Key Laboratory of Wetland Services and Restoration, Institute of Wetland Research, Chinese Academy of Forestry, Beijing, China; 2 Key Laboratory of Ecosystem Network Observation and Modeling, Institute of Geographic Sciences and Natural Resources Research, Chinese Academy of Sciences, Beijing, China; 3 School of Resources and Environmental Engineering, Ludong University, Yantai, China; Tennessee State University, UNITED STATES

## Abstract

Coastal wetlands are considered as a significant sink of global carbon due to their tremendous organic carbon storage. Coastal CO_2_ and CH_4_ flux rates play an important role in regulating atmospheric CO_2_ and CH_4_ concentrations. However, the relative contributions of vegetation, soil properties, and spatial structure on dry-season ecosystem carbon (C) rates (net ecosystem CO_2_ exchange, NEE; ecosystem respiration, ER; gross ecosystem productivity, GEP; and CH_4_) remain unclear at a regional scale. Here, we compared dry-season ecosystem C rates, plant, and soil properties across three vegetation types from 13 locations at a regional scale in the Yellow River Delta (YRD). The results showed that the *Phragmites australis* stand had the greatest NEE (-1365.4 μmol m^-2^ s^-1^), ER (660.2 μmol m^-2^ s^-1^), GEP (-2025.5 μmol m^-2^ s^-1^) and acted as a CH_4_ source (0.27 μmol m^-2^ s^-1^), whereas the *Suaeda heteroptera* and *Tamarix chinensis* stands uptook CH_4_ (-0.02 to -0.12 μmol m^-2^ s^-1^). Stepwise multiple regression analysis demonstrated that plant biomass was the main factor explaining all of the investigated carbon rates (GEP, ER, NEE, and CH_4_); while soil organic carbon was shown to be the most important for explaining the variability in the processes of carbon release to the atmosphere, i.e., ER and CH_4_. Variation partitioning results showed that vegetation and soil properties played equally important roles in shaping the pattern of C rates in the YRD. These results provide a better understanding of the link between ecosystem C rates and environmental drivers, and provide a framework to predict regional-scale ecosystem C fluxes under future climate change.

## Introduction

Carbon dioxide (CO_2_) and methane (CH_4_) are key greenhouse gases (GHGs) that make substantial contributions to global warming [1]. Numerous studies have estimated global wetland CO_2_ and CH_4_ fluxes, but with great uncertainties, mainly due to complicated environmental drivers [2–4]. Coastal wetlands have been recognized as the most vulnerable and sensitive ecosystems, because they act as the ecotone between terrestrial and aquatic ecosystems [5]. Coastal estuary wetlands store at least 430 Tg of carbon (C) with a C sequestration rate of 45 g C m^-2^ yr^-1^, playing an important role in the global carbon cycle as natural carbon pools [6]. The coastal wetland is one of the most important wetland types for understanding C flux dynamics due to the high variations involved with water conditions, sedimentation characteristics, and vegetation types [7]. Coastal wetlands can act as greenhouse gas sinks via C burial, sediment deposition, and plant biomass accumulation, and as greenhouse gas sources through the release of CO_2_ and CH_4_ produced by the decomposition of organic matter [8], so they are of vital importance in governing the atmospheric concentrations of CO_2_ and CH_4_ [9]^.^ However, due to the complicated interaction of environmental factors including vegetation and soil properties, how to disentangle the contributions of multiple drivers to CO_2_ and CH_4_ fluxes in estuary wetland remains unclear.

Vegetation exerts a major influence on C fluxes including net ecosystem exchange (NEE), ecosystem respiration (ER), gross ecosystem productivity (GEP), and CH_4_ flux [10–13]. Niu et al. [14] found that a shift in the coverage of dominant plants could regulate the ecosystem C fluxes including NEE, ER, and GEP. A meta-analysis showed that NEE, ER, and GEP varied with different vegetation types in global coastal wetlands [12]. A previous study demonstrated that the plant biomass of *Arctophila fulva* was a strong predictor of C flux in an arctic tundra wetland [10]. *Spartina alterniflora* could alter the relationship between CH_4_ and electron acceptors, resulting in an increase of CH_4_ flux [11].

Soil properties have been found to be strongly associated with ecosystem C fluxes. Previous studies have reported that soil organic carbon (SOC), dissolved organic carbon (DOC), NH_4_^+^, pH, and salinity all exhibited dominant effects on ecosystem C fluxes [2,15–19]. For example, Ardón et al. [20] identified salinity and hydrology as the most important determinants of GHG fluxes in salt marsh. However, other studies have considered DOC, SOC, and pH as the related factors to predict CH_4_ flux in coastal wetlands [15,16]. Furthermore, the soil water level has also been considered as the main driving factor of regulating CO_2_ and CH_4_ fluxes in estuary and other coastal wetlands [18,19,21,22]. However, the underlying mechanism of regulating ecosystem C fluxes remains unclear.

Spatial structure, which represents underlying effects of the heterogeneity of environmental factors influences the pattern of C fluxes in a different way to biological and environmental factors acting on community and ecosystem [23]. Previous studies have found that soils with similar environmental characteristics have similar microbial communities, which is important for C release through C decomposition and CH_4_ production [24,25]. It was found that spatial heterogeneity imposed strong influence on the variation of soil CO_2_ efflux in tropical riparian ecosystems[4]. However, understandings of the role spatial structure plays in determining ecosystem C fluxes in coastal wetlands is still limited.

The Yellow River Delta (YRD) wetland, which is the largest wetland ecosystem in the warm temperate zone of China, has an obvious dry season (April to June) and rainy season (July to September) [26–28]. Previous studies have reported seasonal variations in ecosystem C fluxes (CO_2_ and CH_4_) based on continuous flux measurements [28–30] and found that dry-season had the second largest contribution to the CO_2_ and CH_4_ emissions in the YRD [29]. Therefore, in this study, we emphasized the relationship between dry-season ecosystem C rates (GEP, ER, NEE, and CH_4_) with vegetation and soil properties and disentangled their contributions to the C rates. Specifically, we compared dry-season ecosystem C rates from 13 locations across three vegetation types at a regional scale in the YRD. The objectives of this study are (1) to test whether soil salinity was the primary determinant affecting ecosystem C rates in the YRD; and (2) to disentangle the contributions of vegetation, soil properties, and spatial structure on dry-season ecosystem C rates at a regional scale.

## Materials and methods

### Site description

**Fig 1:**
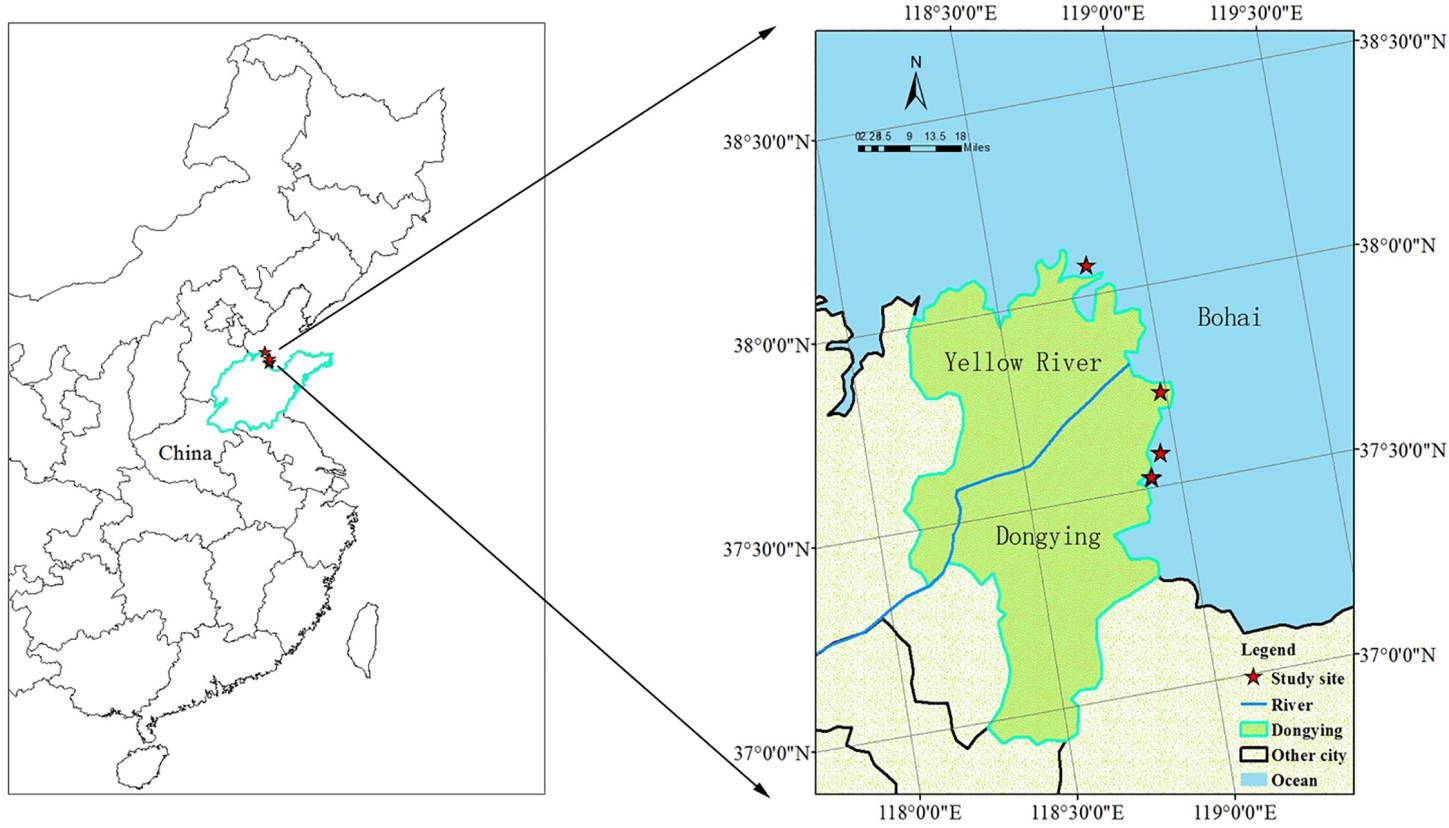
Sample locations at regional scale in coastal zone of Yellow River Delta.

The coastal zone of the Yellow River Delta (Fig 1) has a temperate semi-humid continental monsoon climate [31]. The annual average temperature is 12.9°C. The average annual precipitation is 530–630 mm and the rainfall mostly precipitates from July to September (rainy season). The dry season (April to June) accounts for about 30% of the annual precipitation [28]. The abundant vegetation of this research area includes *Phragmites australis*, *Suaeda heteroptera Kitag*, and *Tamarix chinensis*. Saline soil is the main soil type and the soil texture is sandy loam. Plant biomass and soil properties of each location are listed in Table 1. Locations of samples collected are listed in S1 Table. All necessary permits were obtained for the described field research. We are authorized by the Administrative Office of Yellow River Delta National Nature Reserve to carry out soil and plant collection.

**Table 1:** Sample locations, plant coverage, plant biomass, and soil properties. SWC, soil water content; SOC, soil organic carbon; TP, total phosphorus content; AP, available phosphorus; DOC, dissolved organic carbon; MBC, microbial biomass carbon.

Location	No.	Plant coverage %	Biomass g/m^2^	SWC %, w/w	pH	Salinity ms/cm	SOC g/kg	TP g/kg	AP mg/kg	DOC mg/kg	Ammonia mg/kg	Nitrate mg/kg	MBC mg/kg
Hongguang1	1	30.00	64.32	18.41	8.90	6.80	6.10	0.58	7.36	98.61	4.08	1.63	228.08
Hongguang1	2	10.00	32.64	19.34	8.80	6.10	7.09	0.59	6.89	106.53	1.68	1.52	201.88
Hongguang1	3	20.00	34.36	19.47	8.70	6.40	6.75	0.58	7.08	105.32	1.51	1.38	173.32
Hongguang2	4	40.00	87.52	19.80	8.90	6.70	6.34	0.59	6.60	93.20	1.04	0.73	159.53
Hongguang2	5	20.00	37.96	19.77	8.90	6.20	6.56	0.61	6.60	92.21	1.17	0.95	188.46
Hongguang2	6	16.00	32.68	19.37	8.70	6.48	5.94	0.45	7.36	89.49	1.85	1.18	201.95
Hongguang3	7	20.00	31.52	19.63	8.90	6.85	7.30	0.63	6.89	114.74	4.81	0.91	217.07
Hongguang3	8	16.00	41.32	17.12	9.00	6.88	6.01	0.64	7.27	99.21	2.62	2.13	196.48
Hongguang3	9	18.00	37.40	20.41	8.80	6.36	6.02	0.61	7.17	92.60	1.95	1.54	146.14
Hongguang4	10	58.00	46.88	18.09	8.90	6.91	8.54	0.57	8.61	102.70	3.38	2.90	212.99
Hongguang4	11	50.00	47.40	18.49	8.90	6.92	8.24	0.61	7.27	101.29	2.60	0.92	214.05
Hongguang4	12	22.00	38.12	17.50	8.90	6.90	7.89	0.61	6.98	100.30	2.66	2.05	155.09
Hongguang5	13	62.00	34.00	18.66	9.00	6.93	6.10	0.59	6.69	102.20	2.68	0.94	200.19
Hongguang5	14	52.00	43.96	20.97	8.90	6.86	6.09	0.61	6.89	102.18	4.19	1.18	176.61
Hongguang5	15	40.00	57.68	21.20	8.80	6.35	7.32	0.63	6.31	101.59	3.19	1.55	191.89
Hongguang6	16	15.00	28.64	22.28	8.80	5.28	6.03	0.57	6.60	105.15	6.36	1.35	179.60
Hongguang6	17	15.00	34.24	21.61	8.90	4.76	5.94	0.63	6.60	99.90	8.08	0.94	192.89
Hongguang6	18	25.00	37.00	25.50	8.80	4.55	6.09	0.60	7.65	93.53	4.61	1.09	202.97
Huifuqu1	19	10.00	14.80	18.73	8.90	5.97	6.07	0.57	6.22	89.44	7.12	1.70	171.75
Huifuqu1	20	12.00	12.24	26.28	8.80	5.26	6.54	0.59	8.51	99.51	4.55	1.83	205.10
Huifuqu1	21	16.00	22.76	23.14	8.80	6.25	7.43	0.57	7.36	102.31	3.32	1.36	196.72
Huifuqu2	22	23.00	33.80	24.97	8.70	6.26	7.33	0.61	7.56	98.46	2.48	1.15	232.53
Huifuqu2	23	12.00	19.20	31.84	8.70	5.62	7.99	0.58	9.37	111.82	7.55	1.89	221.85
Huifuqu2	24	20.00	38.08	23.60	8.80	6.14	5.06	0.55	8.42	107.95	4.71	1.74	213.15
Laohekou1	25	80.00	509.60	16.70	8.40	7.07	9.57	0.54	6.41	96.42	3.64	1.65	223.42
Laohekou1	26	80.00	384.27	46.95	8.50	6.97	10.90	0.56	7.84	117.51	3.61	1.15	241.16
Laohekou1	27	22.00	87.67	19.28	8.40	7.09	10.13	0.57	10.42	99.90	2.50	5.13	230.55
Laohekou2	28	38.00	89.51	18.17	8.10	6.83	8.63	0.49	5.83	125.39	3.04	1.34	213.20
Laohekou2	29	23.00	39.96	18.35	8.20	6.97	7.71	0.58	6.03	125.60	7.95	1.36	227.92
Laohekou2	30	25.00	39.64	17.68	8.50	6.94	7.12	0.54	8.80	109.11	4.33	1.71	211.94
Laohekou3	31	20.00	16.76	28.02	8.60	6.43	12.41	0.55	7.75	150.10	28.70	0.97	242.39
Laohekou3	32	20.00	25.04	33.48	8.50	6.81	13.16	0.55	9.28	192.21	60.98	0.67	262.27
Laohekou3	33	20.00	20.16	19.77	8.60	6.43	10.86	0.59	9.75	128.57	6.33	1.43	246.45
Ruhaikou1	34	80.00	216.88	29.01	8.30	3.29	14.77	0.63	12.43	208.86	12.56	0.99	245.77
Ruhaikou1	35	85.00	559.37	29.06	8.10	4.67	19.65	0.63	14.15	109.60	3.76	1.12	245.94
Ruhaikou1	36	85.00	351.59	28.88	8.20	3.00	15.66	0.63	9.85	172.91	4.17	1.62	245.30
Ruhaikou2	37	85.00	525.46	38.63	8.10	3.18	20.93	0.62	12.05	208.06	41.79	0.96	284.31
Ruhaikou2	38	90.00	743.26	39.70	8.00	3.28	27.74	0.70	16.54	166.80	25.14	0.94	289.36
Ruhaikou2	39	0.85	356.79	31.81	8.00	3.07	23.87	0.68	19.61	138.98	10.74	0.67	238.79

### Ecosystem carbon fluxes measurement

Previous studies have reported seasonal variation of ecosystem C fluxes (CO_2_ and CH_4_) based on continuous flux measurements in the Yellow River Delta [29,30], while this study emphasized the relationship between dry-season ecosystem C rates (GEP, ER, NEE, and CH_4_) with plant and soil properties. Therefore, dry-season ecosystem C rates were determined in May 2018. Ecosystem C rates including GEP, ER, NEE, and CH_4_ were measured by the closed chamber method. A 40 × 40 cm square stainless steel frame was inserted into the soil at each location. The CO_2_ and CH_4_ fluxes were measured using a greenhouse gas analyzer (UGGA, LGR, USA) attached to a transparent chamber (0.4 m × 0.4 m × 0.5 m). During the measurements, the chamber was placed on the frame and sealed with water [32], and two small fans ran continuously to mix the air inside the chamber. The relationship between CO_2_ concentration and time was linear during the measurement interval. The CO_2_ flux rates were then determined from the slope of the CO_2_ concentration–time function. The coefficients of determination (*r*^2^) of the linear function were greater than 0.95. Following the measurement of NEE, the chamber was vented for 1–2 min, replaced on the frame, and covered with an opaque cloth to measure ER. Negative NEE values represented net CO_2_ uptake by the ecosystem, and positive NEE values represented net CO_2_ loss. GEP was calculated as the difference between NEE and ER. The CO_2_ and CH_4_ flux measurements were performed between 9:00 am and 14:00 am. Air temperature was determined manually by inserting a digital thermometer onto the transparent chamber when ecosystem CO_2_ fluxes were measured. Soil temperature was measured.by inserting a digital thermometer into soil at the depth of 0–5 cm.

### Plant and soil sampling and element analyses

After the carbon flux measurements, the plant coverage was estimated using a 40 × 40 cm quadrat with a grid size of 4 × 4 cm in each plot, and plants were subsequently harvested for biomass determination. Five soil cores (3.5 cm diameter) were collected randomly from each plot at a 0–15 cm depth and mixed to one composite sample. The samples were passed through a 2 mm sieve and divided into two parts. One part of fresh soil was used for the determination of soil water content (SWC) and the analysis of ammonium (NH_4_^+^), nitrate (NO_3_^-^), microbial biomass carbon (MBC), and dissolved organic carbon (DOC). The other part was air dried for the determination of soil pH, salinity, soil organic carbon (SOC), total phosphorus (TP), and available phosphorus (AP). Soil NH_4_^+^ and NO_3_^-^ concentrations were determined by extraction with 2 M KCl solution followed by colorimetric analysis on a FIAstar 5000 Analyzer (FIAstar 5000 Analyzer, Foss Tecator, Hillerød, Denmark). Soil MBC was estimated by using a chloroform fumigation extraction method [33]. Soil DOC was extracted by adding 50 mL of 0.5 M potassium sulfate to subsamples of 12.5 g of homogenized soil, and by agitating on an orbital shaker at 120 rpm for 1 h. The filtrate was analyzed using a TOC analyzer (multi N/C 3100, Analytik Jena, Germany). The soil pH was determined in a 1:2.5 soil:water solution (w/v), and the soil conductivity was determined as an index of salinity. SOC and TN were analyzed using a C/N analyzer (multi-N/C 3100 Analytik Jena AG, Germany). TP was analyzed by applying the Murphy Riley method following perchloric acid digestion[34] and AP was determined by treatment with 0.5 mol L^-1^ NaHCO_3_ followed by molybdenum blue colorimetry[35] using a spectrophotometer (UV2550, Shimadzu, Japan).

### Statistical analyses

One-way ANOVA with the Duncan test was used to determine the differences of dry-season ecosystem C rates between vegetation types. Stepwise multiple regression was performed using *step* function based on the smallest AIC selection in in R v3.5.0 with the *vegan* package [36].

Unconstrained ordination (principal component analysis, PCA) was used to compare ecosystem carbon rates among plots (*n* = 39). Constrained ordination (redundancy analysis, RDA) was used to represent the relationships between environmental factors (vegetation, soil and space structure), plot patterns, and ecosystem carbon rates [37]. Vegetation type (*S*. *heteroptera*, *P*. *australis*, and *T*. *chinensis*) was considered as a qualitative factor and other environmental factors as quantitative factors.

In order to separate the effects of environmental factors on ecosystem carbon rates, the variation partitioning procedure was conducted. The environmental factors were divided into three groups: (1) vegetation factors including vegetation type, plant biomass, and plant cover; (2) soil factors including soil water content (SWC), soil pH, soil salinity, soil organic carbon (SOC), total phosphorus (TP), available phosphorus (AP), dissolved organic carbon (DOC), microbial biomass carbon (MBC), NH_4_^+^ (ammonia), and NO_3_^-^ (nitrate); (3) spatial structure (*x*, *y*, *xy*, *x*^2^, *y*^2^, *x*^2^*y*, *xy*^2^, *x*^3^, *y*^3^), where nine terms of latitudinal (*x*) and longitudinal (*y*) coordinates were used to calculate a cubic trend surface. Spatial trend surface analysis is one of the quantitative ecological methods used to study the relation between spatial structure and community [23]. PCA, RDA, and variation partitioning analysis were performed in R v3.5.0 with the *vegan* package [36].

## Results

### Variation in ecosystem carbon rates

**Fig 2:**
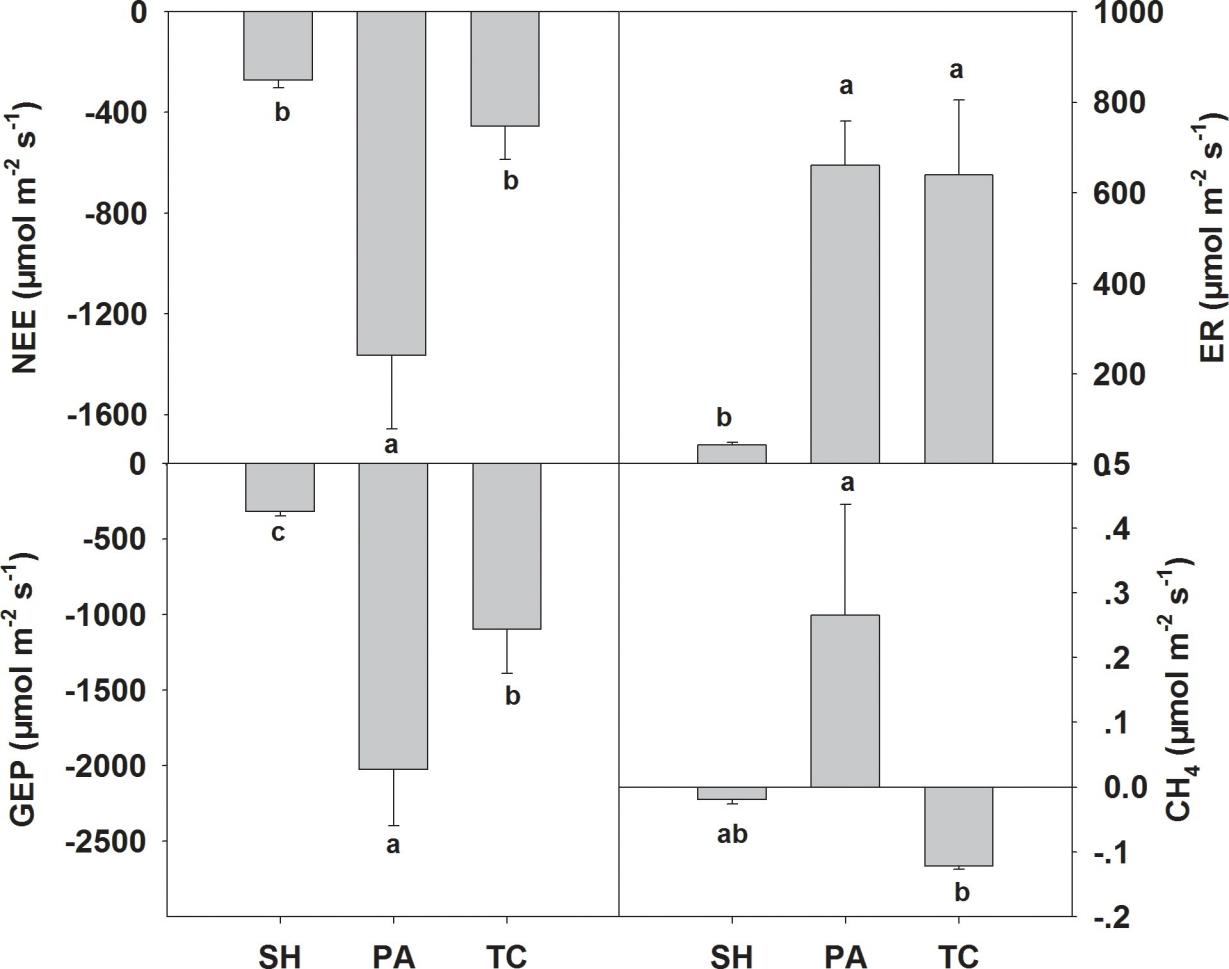
**Ecosystem carbon rates including net ecosystem carbon exchange (NEE, a), ecosystem respiration (ER, b), gross ecosystem productivity (GEP, c) and methane (CH_4_, d) across three vegetation types. SH, *Suaeda heteroptera*; PA, *Phragmites australis*; TC, *Tamarix chinensis*.** Vertical bars represent the standard error of the mean. For SH, PA, and TC *n* = 27, 9, and 3, respectively. Different letters represent significant differences between vegetation types.

The ecosystem carbon rates varied with different vegetation types (Fig 2). Specifically, stands of *P*. *australis* had the highest NEE, ER, and GEP with –1365.3, 660, and –2025.5 μmol m^-2^ s^-1^, respectively, while *S*. *heteroptera* exhibited the lowest values with –272.3, 43.9, and –316.2 μmol m^-2^ s^-1^, respectively. The difference of NEE between *S*. *heteroptera* and *T*. *chinensis* plots was not significant (*P* > 0.05, Fig 2A). Although the NEE of *T*. *chinensis* was significantly lower than that of *P*. *australis*, ER was almost the same as the latter (Fig 2B). Meanwhile, the GEP of *P*. *australis* was much greater than that of *T*. *chinensis*, which resulted in the highest value of NEE in *P*. *australis* (Fig 2A and 2C). With regard to CH_4_ flux, the *S*. *heteroptera* and *T*. *chinensis* plots exhibited –0.019 and –0.12 μmol m^-2^ s^-1^, respectively, and *P*. *australis* exhibited 0.27 μmol m^-2^ s^-1^ (Fig 2D).

**Fig 3:**
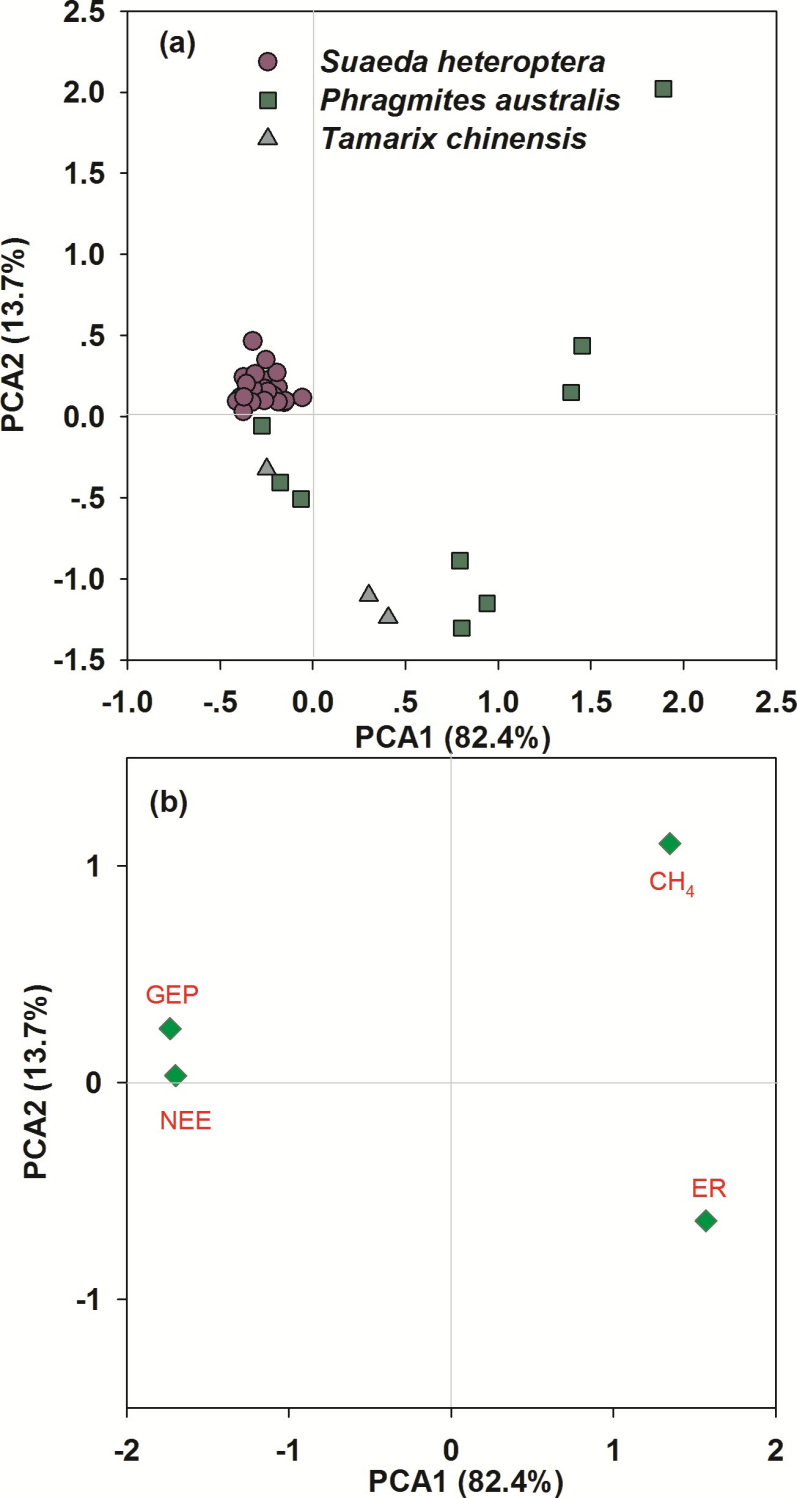
Ordination plots of correspondence analysis (PCA) of all plots and ecosystem carbon rates. (**a**) Ordination plot of 39 plots scores across three vegetation types (*Suaeda heteroptera*, *Phragmites australis*, and *Tamarix chinensis*). (**b**) Ordination plot of four carbon flux rates (GEP, gross ecosystem productivity; NEE, net ecosystem CO_2_ exchange; ER, ecosystem respiration). The ecosystem carbon flux scores are near the points for plots in which they occur with the highest values.

The first axis of PCA ordination explained 82.4% of the variation in the ecosystem carbon rates, mainly reflecting vegetation types (Fig 3A and 3B). *P*. *australis* and *T*. *chinensis* with higher NEE, ER, GEP, and CH_4_ were plotted along the right side of the first axis. *S*. *heteroptera* with lower NEE, ER, GEP, and CH_4_ were concentrated on the left side of the first axis. The second axis of PCA ordination explained 13.7% of the variation in the ecosystem carbon fluxes, mainly associated with soil properties and space variation. The positions of *P*. *australis* and *T*. *chinensis* were separated with the second axis (Fig 3A).

### Relationship between ecosystem carbon rates and environmental factors

**Fig 4:**
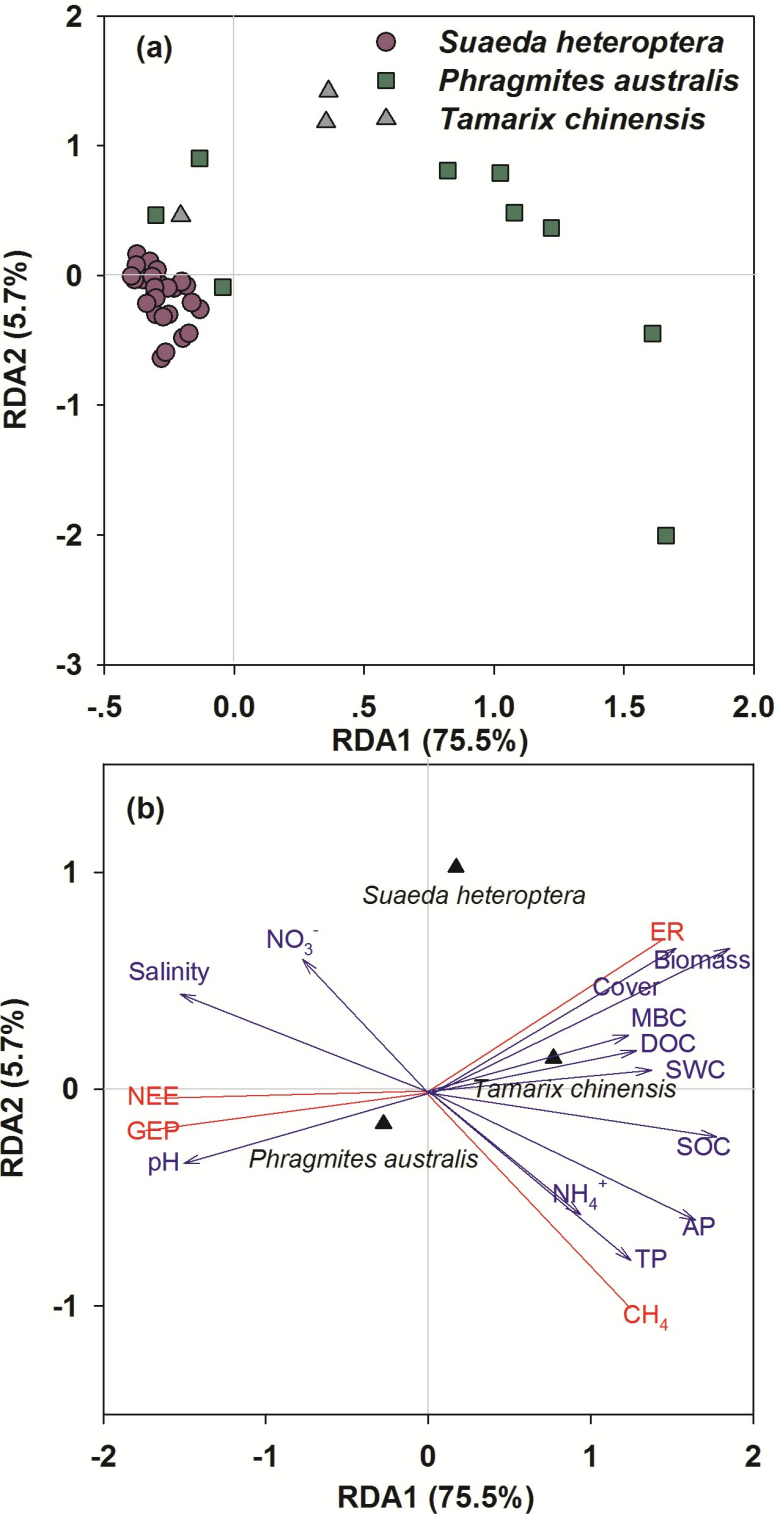
Ordination plots of redundancy analysis (RDA) of all plots and environmental factors. (**a**) Ordination plot of 39 plots scores across three vegetation types. (**b**) Ordination plot of vegetation and soil factors scores in which spatial structure was considered as covariate. Vegetation factors include vegetation type (*Suaeda heteroptera*, *Phragmites australis*, and *Tamarix chinensis*), plant biomass and plant coverage. Soil factors include soil water content (SWC), soil pH, soil salinity, soil organic carbon (SOC), total phosphorus (TP), available phosphorus (AP), dissolved organic carbon (DOC), microbial biomass carbon (MBC), NH_4_^+^ (ammonia), and NO_3_^-^ (nitrate). Vegetation type was plotted as centroids (qualitative factor) and others were plotted as vectors (quantitative factors).

Ecosystem carbon rates across three vegetation types at the regional scale were distinguished by environmental factors with the RDA ordination (Fig 4A and 4B). The first axis described 75.5% of variation in the ecosystem carbon rates, mainly associated with SWC, pH, and salinity. The second axis explained 5.7% of the variation, primarily related to vegetation type, plant biomass, and plant coverage. Soil properties including MBC, DOC, and NO_3_^-^ did not show strong relationships, therefore were not considered as the major factors influencing ecosystem carbon rates (Fig 4B).

Stepwise multiple regression analysis demonstrated that 84% of the variation in NEE could be explained by DOC, plant coverage, and biomass together. SOC, plant coverage, and biomass together contributed for 90% of the spatial variation in ER. Plant coverage and biomass together explained 94% of the variation of GEP. Of the variation of CH_4_ rate, 66% could be attributed to the combination of SOC, salinity and AP (Table 2).

**Table 2:** Results of stepwise multiple regression analysis. Independent variables: vegetation type, plant biomass, plant coverage, soil water content (SWC), soil pH, soil salinity, soil organic carbon (SOC), total phosphorus (TP), available phosphorus (AP), dissolved organic carbon (DOC), microbial biomass carbon (MBC), NH_4_^+^ (ammonia), NO_3_^-^ (nitrate), soil temperature at depth of 5 cm (Ts), and air temperature (Ta). The bold numbers represent significance (*p* < 0.05).

	Variable entered	Parameter estimate	Partial *r*^2^	Probability	AIC
NEE	DOC	–229.28	0.36	**0.000**	411.1
	Coverage	–2.15	0.32	**0.000**	
	Biomass	–256.49	0.16	**0.000**	
	TP	–3.26	0.03	**0.000**	
	AP	–1029.81	0.02	**0.001**	
	NO_3_^-^	–56.62	0.02	**0.004**	
	MBC	58.40	0.01	**0.01**	
	Ta	2.52	0.02	**0.003**	
	Ts	21.31	0.00	0.17	
ER	SOC	18.6624	0.65	**0.0000**	354.97
	Coverage	241.7901	0.16	**0.0000**	
	Biomass	1.4098	0.08	**0.0000**	
	TP	–615.872	0.01	**0.0373**	
	AP	–17.049	0.00	0.3512	
	NH_4_^+^	–4.8788	0.01	0.0550	
	SWC	6.966	0.01	**0.0071**	
	Ts	9.9031	0.01	**0.0068**	
GEP	Coverage	–534.21	0.73	**0.0179**	413.08
	Biomass	–5.14	0.21	**0.0000**	
	AP	–65.55	0.01	**0.0003**	
	NO_3_^-^	103.72	0.01	**0.0187**	
	MBC	1.96	0.00	0.1412	
	Ta	22.61	0.00	0.0729	
CH_4_	SOC	0.2660	0.51	**0.0000**	–146.76
	Coverage	0.0255	0.01	0.2673	
	Biomass	0.1680	0.02	0.1026	
	Salinity	–0.0010	0.06	**0.0107**	
	AP	–0.0349	0.09	**0.0029**	
	NO_3_^-^	0.0704	0.02	0.1184	
	SWC	–0.0526	0.01	0.1899	
	MBC	–0.0036	0.02	0.0948	
	Ta	–0.0020	0.01	0.4252	

### Variation partitioning

**Fig 5:**
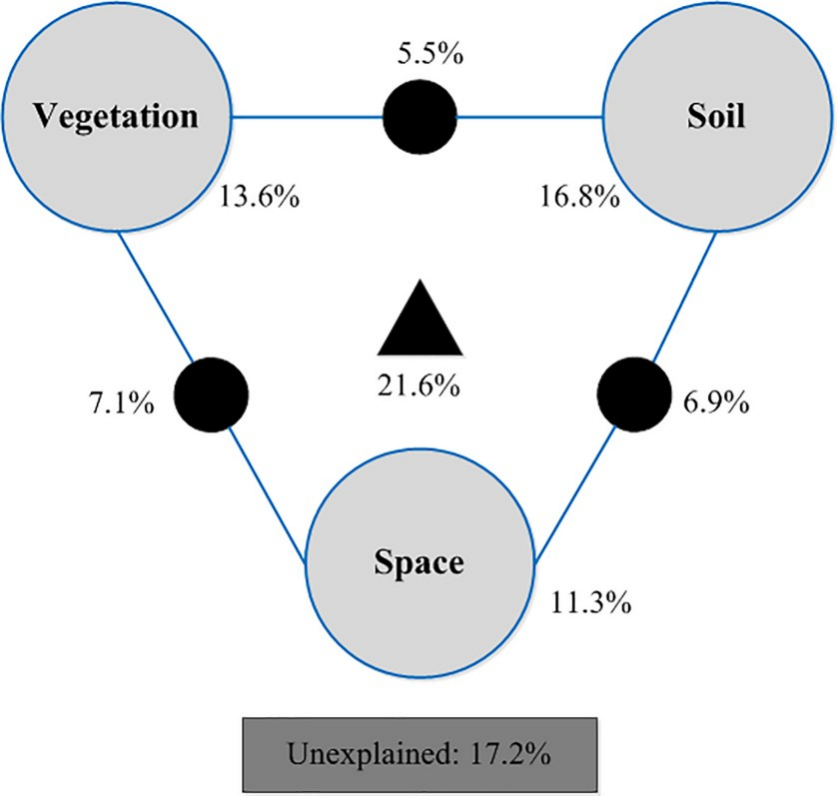
Variation partitioning procedure of ecosystem carbon rates, explained by vegetation factors (vegetation type, plant cover, plant biomass), soil factors (pH, salinity, organic carbon, total phosphorus, available phosphorus, dissolved organic carbon, ammonia, nitrate, water content, microbial biomass carbon), and spatial structure (*x*, *y*, *xy*, *x*^2^, *y*^2^, *x*^2^*y*, *xy*^2^, *x*^3^, *y*^3^: The nine terms in which latitudinal (*x*) and longitudinal (*y*) coordinates were used to calculate a cubic trend surface) factors.

Forward selection of the three groups of environmental factors with RDA suggested that the variation of ecosystem C fluxes was significantly associated with the vegetation (plant biomass) and soil properties (SOC and NH_4_^+^). The variation partitioning results demonstrated that the total explained variation in ecosystem carbon fluxes was 82.8% and the undetermined variation was 17.2% (Fig 5). The vegetation, soil, and spatial factors explained a variation of 13.6%, 16.8%, and 11.3%, respectively, while the largest fraction in the determined variation was the combination of vegetation, soil, and space (21.6%). In addition, the overlap of vegetation and soil, vegetation and space, and soil and space explained a variation of 5.5%, 7.1%, and 6.9%, respectively (Fig 5).

## Discussion

### Effects of vegetation on ecosystem C rates

In the exploration of the primary drivers regulating coastal wetland C rates (NEE, ER, GEP, and CH_4_) and disentangling the relative contributions of multiple environmental factors (vegetation type, plant biomass, coverage, soil pH, salinity, SOC, SWC, TP, AP, DOC, MBC, NH_4_^+^ and NO_3_^-^) and spatial structure on ecosystem C rates, in this study, plant biomass showed as one of the main factors influencing NEE, ER and GEP across three vegetation types at the regional scale.

The mean value of NEE, ER, and GEP of the *P*. *australis* stand was the highest among the three species (Fig 2), suggesting that vegetation plays an important role in regulating ecosystem CO_2_ exchange (Table 2). The role of biotic control of plants on ecosystem CO_2_ exchange has been evaluated in various Chinese coastal wetland ecosystems [6,30,38]. Higher ecosystem CO_2_ exchange rates have been attributed to the higher plant biomass, as plant biomass can regulate the supply of plant litter and hence organic matter decomposition, which in turn affects the rate of CO_2_ production [39]. In this study, plant biomass and coverage were included in the regression models of NEE, ER, and GEP (Table 2). These results suggest that the variations in plant biomass could be one possible reason accounting for the difference in ecosystem CO_2_ exchange among the three vegetation types in the YRD.

It has been proposed that plant biomass could be used to indicate the levels of CH_4_ flux because the litter input provides C resources for the growth of methanogenesis [10,40]. For instance, Andresen et al. [10] reported that CH_4_ flux responded linearly to the biomass increase of *Arctophila fulva*. However, regression results suggested that plant biomass was not the main factor influencing CH_4_ emission in this study (Table 2). The difference of mean CH_4_ rate between the three vegetation types was significant and *P*. *australis* had the highest CH_4_ emissions with the others as a sink of CH_4_ (Fig 2). Differences in the CH_4_ rate among species were likely to be related to a combination of factors such as the growth form [41] and the depth preference among species, which may influence soil temperature [42], methanogenesis, and CH_4_ transport [40]. The high CH_4_ emission rate of *P*. *australis* might be attributed to several reasons. Firstly, large aerenchyma conduits and the presence of this species in relatively deeper water, resulting in the capability of *P*. *australis* roots to extend into CH_4_-rich soils without competition for methanogenic substrates and allowing CH_4_ to be ventilated straight from the soil of these aquatic systems and into the atmosphere [43]. Secondly, high plant biomass and the perennial nature of *P*. *australis* could change soil micro-environment (e.g. higher soil moisture, anaerobic condition) and thus result in high CH_4_ emission[40,43]. These findings suggest that although plant biomass is not the best predictable variable in this coastal wetland, plant species plays an important role in regulating the CH_4_ rate on a community scale.

### Effects of soil properties on ecosystem C rates

Microbiological processes and the roles of microorganisms could be another reason for the variation in ecosystem CO_2_ and CH_4_ rates. Their activities are controlled by several biological, chemical, and physical factors in soil [19,20,22,28,29,44]. Therefore, soil properties including soil organic carbon (SOC), total nitrogen (TN), inorganic nitrogen, total phosphorus (TP), available phosphorus (AP), salinity, and pH are closely related to CO_2_ and CH_4_ fluxes [45]. Previous studies have reported that the variation of ecosystem CO_2_ exchange (NEE, ER and GEP) is associated with the shift in sediment pH and salinity [46]. This could be attributed to the high sensitivity of soil microbes to changes in pH and salinity [47]. Previous researches also demonstrated that ecosystem carbon fluxes were regulated by soil N and P concentration and nutrient stoichiometry[48,49]. The present study showed that NEE had a significant relationship with DOC, plant coverage, plant biomass, TP, AP, NO_3_^-^, MBC, and Ta; ER had a significant relationship with SOC, plant coverage, plant biomass, TP, and soil water content (SWC); and GEP was closely related with plant biomass, plant coverage, AP, and NO_3_^-^. It is worth noting that in contrast to previous studies, NEE, ER, and GEP were not significantly associated with soil pH and salinity in this coastal zone of the YRD.

Methanogenic bacteria are pH sensitive and most of them grew over a relatively narrow pH range of about 6–8, with an optimum pH of 7.7 for methane emission in coastal wetlands [50]. In this study, pH ranged from 8.0 to 9.0, which is not an optimum pH for methane emission. The CH_4_ rate in coastal wetlands was enhanced at low salinity due to the intense oxidation or alleviation of competition by more efficient sulfate- and nitrate-reducing bacteria than methanogenic bacteria and a previous study has shown a strong negative relationship between CH_4_ emission and salinity [45]. In this study, except for SOC and salinity, the CH_4_ rate was significantly associated with AP, which was probably due to the intense anthropogenic nutrient inputs[51].

### Future needed studies and implication for dry-season ecosystem C rates in coastal wetlands under climate change

It should be noted that only the dry-season ecosystem C rates (GEP, ER, NEE and CH_4_) were measured in this study, which hampered calculating the yearly C fluxes. However, the conclusions drawn from the results of the relationship between dry-season ecosystem C rates and vegetation and soil properties were not compromised. Further studies should investigate the long-term ecosystem C rates and their relationships with vegetation and soil factors, which provide the foundation in which to explore the mechanism of the variation of ecosystem C fluxes.

In our study area, the vegetation was not homogeneous and the community types comprised of the native species *P*. *australis*, *S*. *salsa*, and *T*. *chinensis*. The spatial distribution pattern of vegetation was mostly identified as patches of these species or mud flats (bare soil) on the scale of meters to tens of meters due to the soil salt content gradient [6]. Wetland vegetation is the carrier of coastal C sink, which is of vital importance to mitigate climate change [5]. In addition, we did not sample the underground biomass of these stands to explain the variation of ecosystem C rates. These parameters warrant further investigation.

Previous study has demonstrated that the average annual precipitation has decreased with a rate of 4.5 mm yr^-1^ and the annual air temperature has increased by 1.7°C over the past 55 years in the YRD [28]. This shift of precipitation and air temperature might induce drier and hotter seasons in the YRD in the future, which has a great possibility to increase ER, thus greater C loss from this coastal wetland. These findings have important implications for predicting ecosystem C rates to changes in precipitation and air temperature under climate change [52].

## Conclusions

In conclusion, this study comprehensively investigated the regulation of ecosystem C flux rates under vegetation and soil property gradients in the coastal zone of the Yellow River Delta. This study showed that a combination of biotic and abiotic predictors, e.g., vegetation and soil, explained the majority of the variation in the investigated dry-season ecosystem C rates in the coastal zone of the Yellow River Delta. Plant biomass was found to be the main factor explaining all of the investigated carbon rates (GEP, ER, NEE, and CH_4_), while soil organic carbon was shown to be the most important for explaining the variability in the processes of carbon release to the atmosphere, i.e., ER and CH_4_. Vegetation and soil properties played equally important roles in shaping the pattern of C rates through variation partition analysis. The results of this research provide a better understanding of the link between ecosystem C rates and environmental drivers, and provide a good basis to predict regional‐scale ecosystem C fluxes under future climate change.

## Supporting information

S1 Table Sample locations, coordinates, and vegetation types. (DOCX)
