# Supplementary material for: Plant biomass and soil organic carbon are main factors influencing dry-season ecosystem carbon rates in the coastal zone of the Yellow River Delta
Source: PLoS One. 2019 Jan 14;14(1):e0210768. doi: 10.1371/journal.pone.0210768 (PMC6331112; doi:10.1371/journal.pone.0210768)
Supplement: S1 Table — (DOCX) [file pone.0210768.s001.docx]

**S1 Table.** Sample locations, coordinates ,and vegetation types.

| Location | No. | Latitude | Longitude | Vegetation type |
| --- | --- | --- | --- | --- |
| Hongguang1 | 1 | 37.525353N | 118.90849E | *Suaeda heteroptera* |
| Hongguang1 | 2 | 37.52537 N | 118.9089 E | *Suaeda heteroptera* |
| Hongguang1 | 3 | 37.525387 N | 118.90929 E | *Suaeda heteroptera* |
| Hongguang2 | 4 | 37.525668 N | 118.90802 E | *Suaeda heteroptera* |
| Hongguang2 | 5 | 37.525702 N | 118.90836 E | *Suaeda heteroptera* |
| Hongguang2 | 6 | 37.525736 N | 118.90878 E | *Suaeda heteroptera* |
| Hongguang3 | 7 | 37.525893 N | 118.9081 E | *Suaeda heteroptera* |
| Hongguang3 | 8 | 37.525931 N | 118.90847 E | *Suaeda heteroptera* |
| Hongguang3 | 9 | 37.525961 N | 118.90886 E | *Suaeda heteroptera* |
| Hongguang4 | 10 | 37.526323 N | 118.90763 E | *Suaeda heteroptera* |
| Hongguang4 | 11 | 37.526348 N | 118.90797 E | *Suaeda heteroptera* |
| Hongguang4 | 12 | 37.526416 N | 118.90828 E | *Suaeda heteroptera* |
| Hongguang5 | 13 | 37.52650 N | 118.9080 E | *Suaeda heteroptera* |
| Hongguang5 | 14 | 37.526565 N | 118.9080 E | *Suaeda heteroptera* |
| Hongguang5 | 15 | 37.526595 N | 118.90832 E | *Suaeda heteroptera* |
| Hongguang6 | 16 | 37.525144 N | 118.90918 E | *Suaeda heteroptera* |
| Hongguang6 | 17 | 37.525165 N | 118.90953 E | *Suaeda heteroptera* |
| Hongguang6 | 18 | 37.525183 N | 118.90992 E | *Suaeda heteroptera* |
| Huifuqu1 | 19 | 37.58280 N | 118.9510E | *Suaeda heteroptera* |
| Huifuqu1 | 20 | 37.582607 N | 118.95094 E | *Suaeda heteroptera* |
| Huifuqu1 | 21 | 37.58242 N | 118.95084 E | *Suaeda heteroptera* |
| Huifuqu2 | 22 | 37.582756 N | 118.95122 E | *Suaeda heteroptera* |
| Huifuqu2 | 23 | 37.58256 N | 118.95113 E | *Suaeda heteroptera* |
| Huifuqu2 | 24 | 37.582288 N | 118.95101 E | *Suaeda heteroptera* |
| Laohekou1 | 25 | 38.076017 N | 118.81435 E | *Tamarix chinensis* |
| Laohekou1 | 26 | 38.075839 N | 118.81434 E | *Tamarix chinensis* |
| Laohekou1 | 27 | 38.075535 N | 118.81431 E | *Tamarix chinensis* |
| Laohekou2 | 28 | 38.076059 N | 118.8141 E | *Phragmites australis* |
| Laohekou2 | 29 | 38.075751 N | 118.81408 E | *Phragmites australis* |
| Laohekou2 | 30 | 38.075434 N | 118.81406 E | *Phragmites australis* |
| Laohekou3 | 31 | 38.076093 N | 118.8139 E | *Suaeda heteroptera* |
| Laohekou3 | 32 | 38.075734 N | 118.81389 E | *Suaeda heteroptera* |
| Laohekou3 | 33 | 38.075404 N | 118.81391 E | *Suaeda heteroptera* |
| Ruhaikou1 | 34 | 37.731367 N | 118.98476 E | *Phragmites australis* |
| Ruhaikou1 | 35 | 37.731427 N | 118.9843 E | *Phragmites australis* |
| Ruhaikou1 | 36 | 37.731588 N | 118.98385 E | *Phragmites australis* |
| Ruhaikou2 | 37 | 37.7311 N | 118.98473 E | *Phragmites australis* |
| Ruhaikou2 | 38 | 37.731308 N | 118.98435 E | *Phragmites australis* |
| Ruhaikou2 | 39 | 37.731376 N | 118.98399 E | *Phragmites australis* |
